# Bilateral Meningo-Cortical Involvement in Anti-myelin Oligodendrocyte Glycoprotein-IgG Associated Disorders: A Case Report

**DOI:** 10.3389/fneur.2021.670349

**Published:** 2021-05-14

**Authors:** Guozhong Ma, Jinzhao He, Yan Li, Yan Xu, Yunxin Hu, Fang Cui

**Affiliations:** ^1^Department of Neurology, Heyuan People's Hospital, Heyuan, China; ^2^Department of Neurology, Guangdong 999 Brain Hospital, Guangzhou, China; ^3^Department of Neurology, Hainan Hospital of Chinese People's Liberation Army General Hospital, Sanya, China

**Keywords:** anti-myelin oligodendrocyte glycoprotein-IgG associated disorders, bilateral, meningo-cortical involvement, magnetic resonance imaging feature, case report

## Abstract

Cortical T2-weighted fluid-attenuated inversion recovery (FLAIR)-hyperintense lesions in anti-myelin oligodendrocyte glycoprotein (MOG)-associated encephalitis with seizures (FLAMES) are mostly unilateral and rarely spread to the bilateral cortex and meninges. We describe a case of MOG-immunoglobulin G (IgG) associated disorder (MOGAD) in a 39-year-old male with bilateral meningo-cortical involvement. The patient was hospitalized for epilepsy, fever, and headache. The initial MRI revealed abnormalities in the sulci of the bilateral frontal, temporal, and parietal lobes. He was considered to have infectious encephalitis and given empiric antibiotic and antiviral therapy, which were ineffective. His condition rapidly improved after the patient was switched to high-dose immunoglobulin therapy. No tests supported the presence of central nervous system (CNS) infections or autoimmune encephalitis. The second and third MRI scans showed reduced but still clearly observable meningo-cortical lesions. The patient was discharged without a definite diagnosis, but reported severe left vision impairment 25 days later. A fourth MRI showed signs typical of demyelinating CNS disease in addition to the original meningo-cortical lesions. The patient's symptoms were initially relieved by low-dose corticosteroid therapy, but they eventually returned, and he was re-admitted. The original lesions were diminished on the fifth MRI scan, but new lesions had developed in the deep white matter. A positive cell-based assay for MOG-IgG in serum confirmed MOGAD. The patient received high-dose corticosteroid treatment followed by an oral methylprednisolone taper, and his visual acuity gradually improved. The sixth and final MRI showed substantial decreases in the original lesions without new lesion formation. This unique case presents the complete diagnosis and treatment process for MOGAD with bilateral meningo-cortical involvement and may provide a reference for prompt diagnosis.

## Introduction

Myelin oligodendrocyte glycoprotein (MOG)-immunoglobulin G (IgG) associated disorder (MOGAD) magnetic resonance imaging (MRI) findings often involve the white matter, thalamus, pons, optic nerve, and spinal cord (1, 2). Notably, the phenotype of unilateral fluid-attenuated inversion recovery (FLAIR)-hyperintense lesions in anti-myelin oligodendrocyte glycoprotein (MOG)-associated encephalitis with seizures (FLAMES) has distinct radiographic manifestations (3) that are different from other common phenotypes. To date, only a few cases of FLAMES have been reported in the literature, and there are limited longitudinal MRI data regarding this entity. This case report covers the complete trajectory of MOGAD findings from initial meningo-cortical involvement to consequent MRI changes.

## Case Presentation

**Figure 1:**
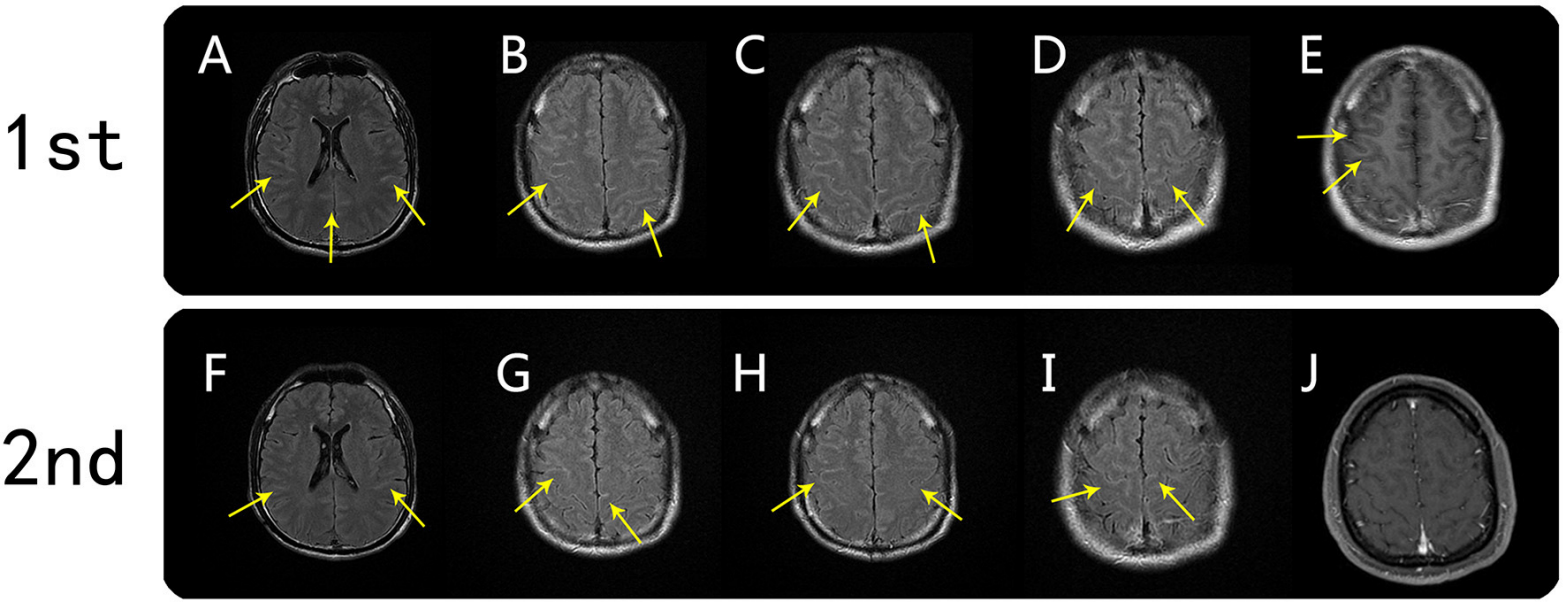
Brain MRI findings from the first and second scans. First MRI scan (on day 2): axial FLAIR hyperintensity was seen in the sulci of the bilateral frontal, temporal, and parietal lobes (**A–D**, arrows). Corresponding meningo-cortical enhancement was also seen on axial T1-weighted postgadolinium-enhanced images (**E**, arrow). Second MRI scan (on day 7): meningo-cortical lesions on axial FLAIR were mildly regressed, but abnormalities were still observed (**F–I**, arrows). T1-weighted postgadolinium-enhanced image showed a corresponding meningo-cortical lesion with no enhancement **(J)**.

**Figure 2:**
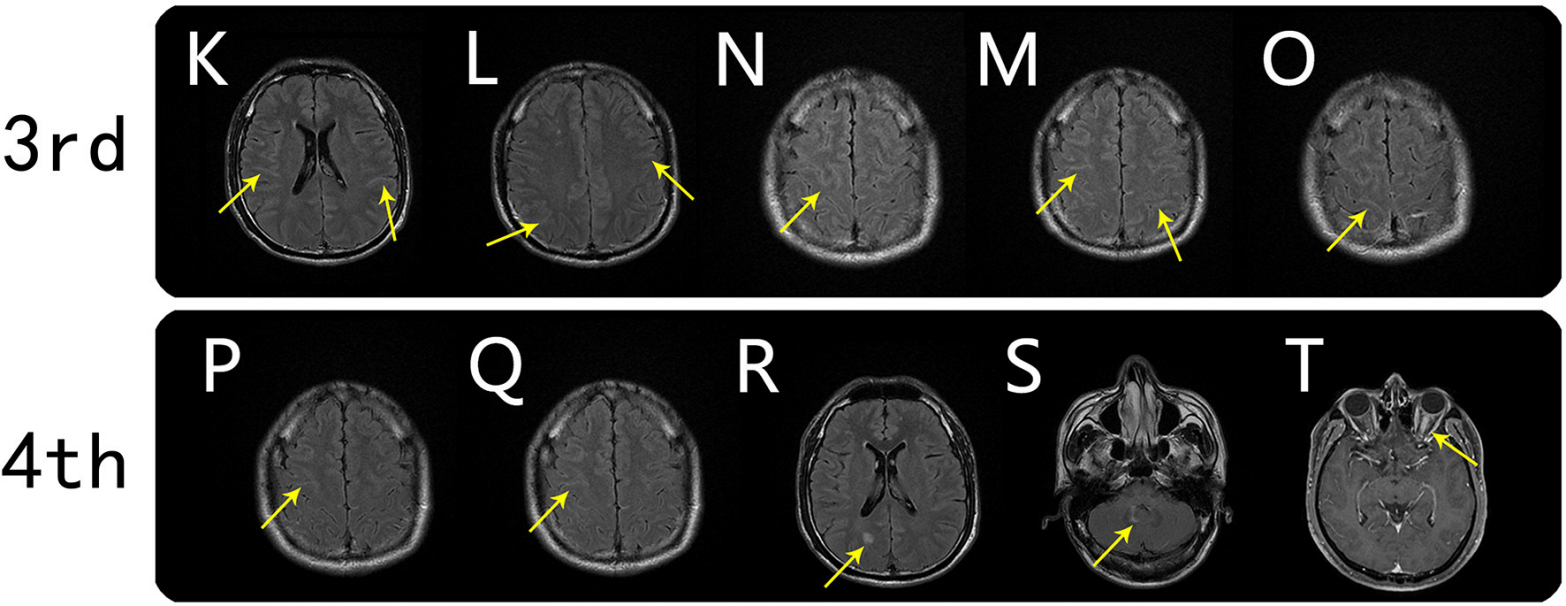
Brain MRI findings from the third and fourth scans. Third MRI scan (on day 22): axial FLAIR showed reduced meningo-cortical lesions that were still clearly observable (**K–O**, arrows). Fourth MRI scan (on day 37): axial FLAIR showed that the meningo-cortical lesions were further resolved (**P** and **Q**, arrows), but new lesions were present in the right parietal cortex and right cerebellar dentate nucleus (**R** and **S**, arrows). T1-weighted postgadolinium-enhanced image showing left optic nerve thickening and obvious enhancement (**T**, arrow).

On June 16, 2020, a 39-year-old male patient was admitted to our hospital following 2 days of seizures that were preceded by 15 days of fever and headache. The patient denied having any prior psychiatric or other diseases. His neck was slightly stiff, and there were suspiciously positive bilateral Kernig's signs. On day 2 of hospitalization, the initial MRI examination showed FLAIR hyperintensities in the cortex and meninges of the bilateral frontal, temporal, and parietal lobes; the abnormalities were also clearly visualized on T1-weighted post-gadolinium-enhanced images (Figure 1). The patient was considered to have developed a central nervous system (CNS) infection and was given empiric antibiotic and antiviral therapy, but his condition steadily worsened, suggesting that there may be other underlying etiologies (e.g., autoimmune encephalitis or tuberculous meningoencephalitis). After considering the pros and cons, we administered high-dose immunoglobulin treatment, and the patient's symptoms rapidly improved. The second MRI examination on day 8 demonstrated mildly regressed meningo-cortical lesions, but abnormalities were still observed (Figure 1). No other test results (listed in Table 1) supported the existence of autoimmune encephalitis or a CNS infection. We did not prescribe corticosteroid therapy due to concern about unknown infections. After further observation, the third MRI examination conducted on day 24 showed that the meningo-cortical lesions had decreased but were still clearly observable (Figure 2). At that time, the patient exhibited good recovery except for mild palpitations and insomnia, and he was discharged on day 30 of hospitalization without a definite diagnosis.

**Table 1:**
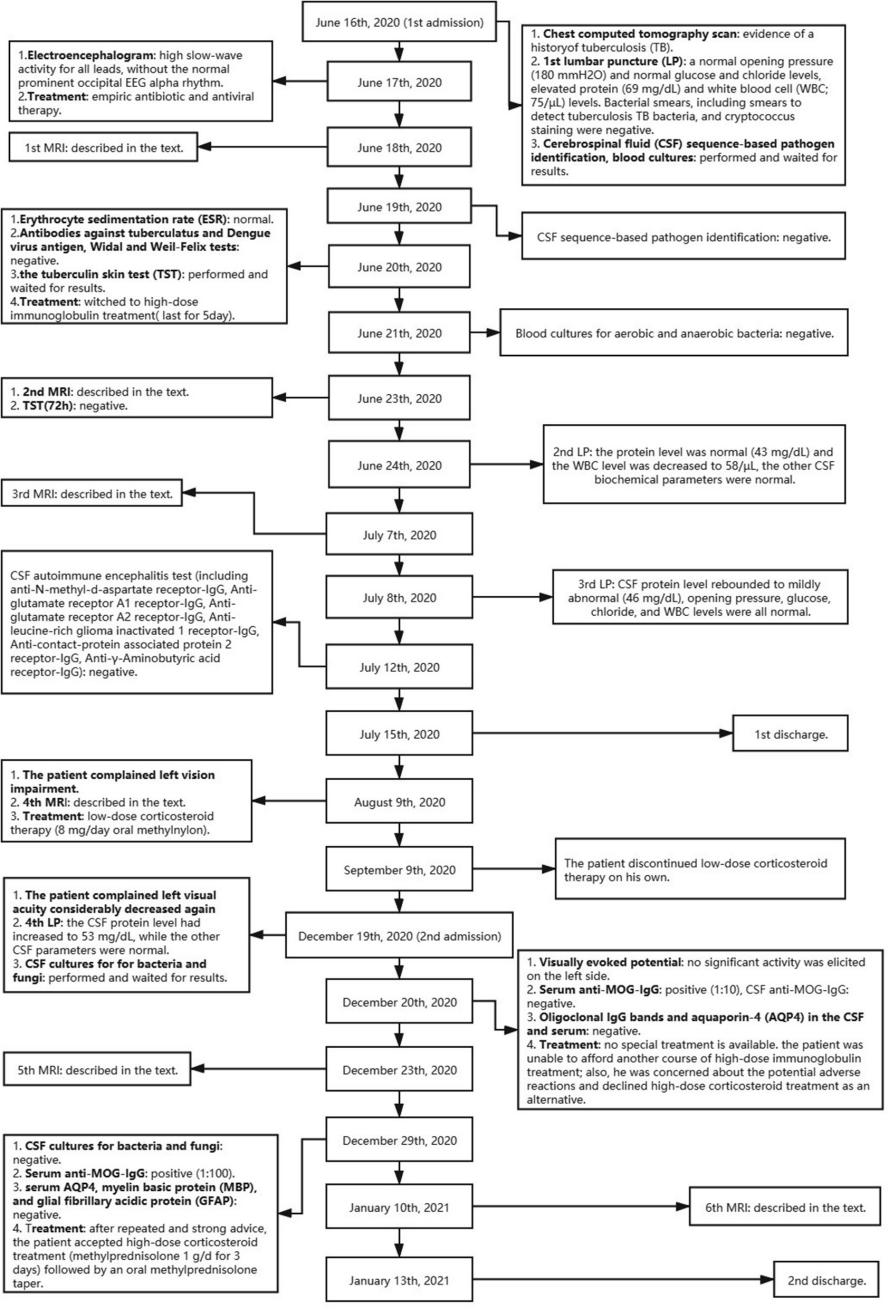
Timeline of the patient's lab data and cerebrospinal fluid results.

**Figure 3:**
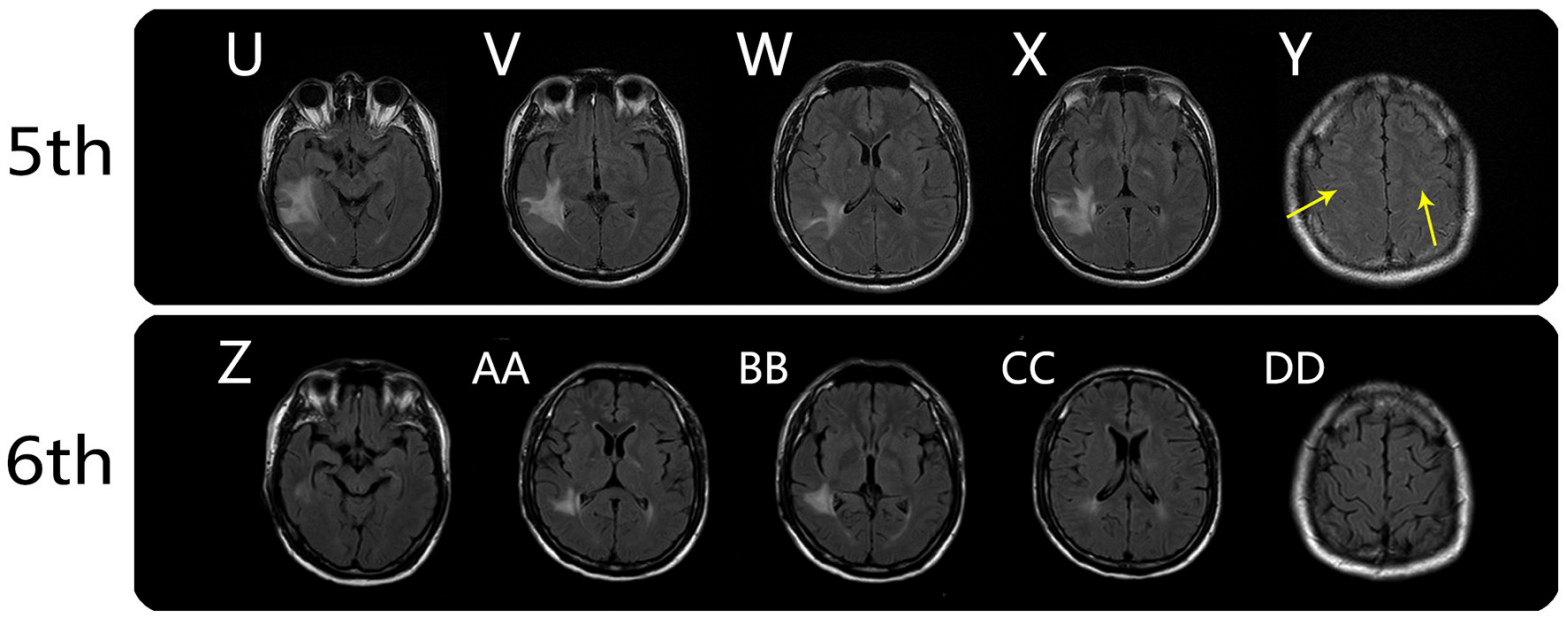
Brain MRI findings from the fifth and sixth MRI scans. Fifth MRI scan (on day 132): axial FLAIR showed persistent meningo-cortical hyperintensity (**Y**, arrows) and new lesions around the temporal lobe, posterior limb of internal capsule (right), and left trigone of the lateral ventricle **(U–X)**. Sixth MRI scan (on day 148): axial FLAIR showed substantial reductions in the original lesions without new lesion formation (**Z**, **AA**, **BB**, and **CC**), and the meningo-cortical FLAIR hyperintensity disappeared **(DD)**.

On August 9, 2020, he was followed-up at our outpatient clinic and reported that the vision in his left eye was severely impaired. This symptom first manifested ~1 month earlier, but it was mild and he did not seek medical help. A fourth MRI examination was immediately conducted and showed that the meningo-cortical lesions were reduced compared, but new lesions were present in the right parietal lobe, right cerebellar dentate nucleus, and left optic nerve (Figure 2). At this point, we realized that a demyelinating CNS disease was responsible for the symptoms. The patient refused hospital re-admission, further examinations, or further high-dose immunoglobulin therapy due to economic reasons, so we prescribed low-dose corticosteroid therapy. The patient's visual acuity was considerably improved after treatment, which he decided to discontinue after ~1 month. On December 19, 2020, the patient complained that his left visual acuity had again deteriorated, and he was re-admitted to the hospital. A fifth MRI examination showed that the original lesions were diminished, but new lesions had developed around the deep white matter (Figure 3). A cell-based assay for serum anti-MOG-IgG was positive, and the final diagnosis was MOGAD. The patient accepted high-dose corticosteroid treatment, and his left visual acuity gradually improved. The sixth and final MRI examination was conducted on January 8, 2021 and showed substantial reductions in the original lesions without new lesion formation (Figure 3). The patient was discharged on January 13, 2021. At the time of writing, he had remained in generally good condition.

In addition to the above MRI examinations, the patient also underwent a series of other tests at different time points that were crucial for diagnosis. The relevant details are listed in the timeline in Table 1.

## Discussion

In 2017, Ogawa et al. (4) first reported a rare MOGAD phenotype with unique unilateral cortical encephalitis properties. Budhram et al. (3) more thoroughly characterized these unique clinico-radiographic syndrome and referred to this entity as FLAMES. Although initially described as a unilateral cortical encephalitis, it can also manifest as bilateral cortical lesions with or without leptomeningeal involvement (3, 5) or even isolated unilateral leptomeningeal enhancement (3, 5). FLAMES is more likely to be misdiagnosed as other CNS diseases such as viral meningitis, carcinomatous meningitis, or subarachnoid hemorrhage. In our case, the patient initially presented with fever, headache, and cerebrospinal fluid findings similar to those associated of viral meningitis. We were unaware of the possibility of bilateral meningo-cortical MOGAD manifestations, which delayed the diagnosis. Given that the FLAMES phenotype is highly steroid-responsive, increased knowledge and appreciation of these symptoms is critical to facilitate timely treatment.

Here we described a case of MOGAD presenting with the MRI feature of bilateral meningo-cortical involvement. The exact correlations between meningeal and cortical involvement are not fully clear, but there are two possible mechanisms. One suggests that primarily meningeal lesions spread to the cortex, while the other proposed that both meningeal and cortical lesions appear simultaneously (3, 8). Unlike the other common phenotype of MOGAD, cases involving cortical lesions do not exhibit distinct pathologic demyelination features and have only mild inflammatory changes (6, 7). One perspective is that MOGAD with cortical lesions should be considered as a new disease associated with anti-MOG antibodies (6). Other recent studies have indicated that anti-MOG antibodies may not be directly associated with the cortical lesion phenotype or may not even be involved in this phenotype (4, 6). Although no evidence of other auto-antibodies has been detected in FLAMES-related phenotypes so far, an unknown auto-antibody might be involved in disease pathogenesis (4, 8).

## Data Availability Statement

The original contributions presented in the study are included in the article/Supplementary Material, further inquiries can be directed to the corresponding author/s.

## Ethics Statement

Written informed consent was obtained from the individual for the publication of any potentially identifiable images or data included in this article.

## Author Contributions

GM, YH, YX, and FC designed the diagnostic and treatment plans. GM, JH, and YL drafted the manuscript. GM and FC revised the manuscript draft. GM, JH, YH, and YX generated the figures. All authors approved the submitted version of the manuscript.

## Conflict of Interest

The authors declare that the research was conducted in the absence of any commercial or financial relationships that could be construed as a potential conflict of interest.
