# Supplementary material for: Bilateral Meningo-Cortical Involvement in Anti-myelin Oligodendrocyte Glycoprotein-IgG Associated Disorders: A Case Report
Source: Front Neurol. 2021 May 14;12:670349. doi: 10.3389/fneur.2021.670349 (PMC8160241; doi:10.3389/fneur.2021.670349)
Supplement: Supplementary file 1 [file Data_Sheet_1.pdf]

CARE Checklist of information to include when writing a case report

(cc) BY-NC-ND

Topic	Item	Checklist item description	Reported on Line
**Title**	1	The diagnosis or intervention of primary focus followed by the words "case report"	Page1, title, line75
**Key Words**	2	2 to 5 key words that identify diagnoses or interventions in this case report, including "case report"	Page1, key words
**Abstract (no references)**	3a	Introduction: What is unique about this case and what does it add to the scientific literature?	Page1, abstract, line109-111
	3b	Main symptoms and/or important clinical findings	Page1, abstract, line 88-89
	3c	The main diagnoses, therapeutic interventions, and outcomes	Page1, abstract, line 90-109
	3d	Conclusion—What is the main "take-away" lesson(s) from this case?	Page4, discussion, line454-464
**Introduction**	4	One or two paragraphs summarizing why this case is unique (**may include references**)	Page1, abstract, line109-111
**Patient Information**	5a	De-identified patient specific information	Yes
	5b	Primary concerns and symptoms of the patient	Page2, case presentation, line134-135
	5c	Medical, family, and psycho-social history including relevant genetic information	Page2, case presentation, line136
	5d	Relevant past interventions with outcomes	Page2, case presentation, line133-135
**Clinical Findings**	6	Describe significant physical examination (PE) and important clinical findings	Page2, case presentation, line 136-137
**Timeline**	7	Historical and current information from this episode of care organized as a timeline	Page3, table 1
**Diagnostic Assessment**	8a	Diagnostic testing (such as PE, laboratory testing, imaging, surveys).	Page2-4, case presentation
	8b	Diagnostic challenges (such as access to testing, financial, or cultural)	Page2, case presentation, line 142-172, line 194-196
	8c	Diagnosis (including other diagnoses considered)	Page2, case presentation, Line142-143, line 192-193, line 203-204
	8d	Prognosis (such as staging in oncology) where applicable	Page4, case presentation, line392-393
**Therapeutic Intervention**	9a	Types of therapeutic intervention (such as pharmacologic, surgical, preventive, self-care)	Page2, case presentation
	9b	Administration of therapeutic intervention (such as dosage, strength, duration)	Page2, case presentation
	9c	Changes in therapeutic intervention (with rationale)	Page2, case presentation
**Follow-up and Outcomes**	10a	Clinician and patient-assessed outcomes (if available)	Page4, case presentation, line392-393
	10b	Important follow-up diagnostic and other test results	Page2-4, case presentation
	10c	Intervention adherence and tolerability (How was this assessed?)	Page2, case presentation, line197-198

**10d** Adverse and unanticipated events			Page2-3, case presentation
**Discussion**	**11a**	A scientific discussion of the strengths AND limitations associated with this case report	Page 4-5, discussion
	**11b**	Discussion of the relevant medical literature **with references**	Page 4-5, discussion
	**11c**	The scientific rationale for any conclusions (including assessment of possible causes)	Page4-5, discussion
	**11d**	The primary "take-away" lessons of this case report (without references) in a one paragraph conclusion	Page4-5, discussion
**Patient Perspective**	**12**	The patient should share their perspective in one to two paragraphs on the treatment(s) they received	Page2, case presentation, line185-186, line 194-195
**Informed Consent**	**13**	Did the patient give informed consent? Please provide if requested	**Yes** ☑ **No** ☐
